# Pro-cognitive effects of 5-HT4 receptor agonism in individuals with remitted depression

**DOI:** 10.1017/S0033291726104450

**Published:** 2026-06-15

**Authors:** Angharad N. de Cates, Sorcha Hamilton, Anutra Guru, Merethe Blandhol, Michael Colwell, Philip J. Cowen, Meghan Simmons, Bailey Jones, Catherine J. Harmer, Susannah E. Murphy

**Affiliations:** 1 Institute for Mental Health, University of Birmingham, Edgbaston, Birmingham, UK; 2 University Department of Psychiatry, Warneford Hospital, University of Oxford, Oxford, UK; 3 Oxford Health NHS Foundation Trust, Warneford Hospital, Oxford, UK; 4 Oxford Centre for Human Brain Activity, Oxford University Centre for Integrative Neuroimaging, Department of Psychiatry, University of Oxford, Oxford, UK

**Keywords:** cognition, procognitive, prucalopride, serotonin, 5-HT4

## Abstract

**Background:**

Cognitive impairment is a common and persistent feature of depression, yet it remains poorly understood and inadequately treated. Preclinical and human studies suggest that stimulating 5-HT_4_ receptors (5-HT_4_R) enhances neuroplasticity and improves cognition. This novel study examines the cognitive effects of 5-HT_4_R agonism in adults with a history of recurrent depression.

**Methods:**

Fifty participants who were not currently depressed but had experienced at least two previous episodes of depression were randomized in a double-blind design either to prucalopride (2 mg daily, titrated from 1 mg) or to placebo for 7–10 days. Participants completed self-report questionnaires and a task battery at baseline and post-intervention assessing declarative memory, working memory, emotional processing, and executive function.

**Results:**

Compared to placebo, prucalopride significantly improved word recall on an auditory verbal learning task and was associated with faster response times on a complex working memory task without loss of accuracy. It also improved the accurate recognition of rapidly presented facial expressions. Composite analysis of non-emotional tasks identified that the prucalopride group participants post-intervention were faster and more accurate than at baseline compared to those receiving placebo. Prucalopride had minimal effects on affective cognition, consistent with previous findings. Cognitive improvements were independent of baseline mood symptoms or self-reported cognitive difficulties.

**Conclusions:**

Short-term 5-HT_4_R agonism improved performance on multiple objective cognitive measures in individuals with remitted depression. These findings replicate our previous results in healthy volunteers showing a pro-cognitive effect of prucalopride and support a role for 5-HT_4_Rs as a promising target for cognitive enhancement in mood disorders.

## Introduction

Cognitive impairment is a common feature of depression, with significant personal and societal costs. These symptoms are poorly addressed by first-line antidepressant treatments (Millan et al., 2012). Around 80% of individuals experience cognitive difficulties during a current episode of depression (Conradi, Ormel, & de Jonge, 2011), and over 40% continue to report subjective cognitive problems even after other symptoms of depression have resolved (McClintock et al., 2011; Rock, Roiser, Riedel, & Blackwell, 2014; Semkovska et al., 2019). Despite their prevalence and impact on functional recovery, treatment options targeting cognitive dysfunction in depression remain limited (Halahakoon & Roiser, 2016). Successive episodes of depression appear to lead to a deterioration in cognition (Kriesche, Woll, Tschentscher, Engel, & Karch, 2023; Semkovska et al., 2019; Varghese, Frey, Schneider, Kapczinski, & de Azevedo Cardoso, 2022). Cognitive impairment is associated with a range of negative long-term outcomes including risk of relapse (Halahakoon, Lewis, & Roiser, 2019), and therefore, targeting cognitive dysfunction in remitted depression may provide an opportunity to reduce the risk of relapse. This highlights the need for novel therapeutic strategies to address cognitive symptoms and support long-term recovery.

One promising target is the serotonin 4 receptor (5-HT_4_R). A robust body of preclinical evidence demonstrates that 5-HT_4_R agonists rapidly improve performance on tasks of learning and memory (Fontana, Daniels, Wong, Clark, & Eglen, 1997; Hagena & Manahan-Vaughan, 2017; King, Marsden, & Fone, 2008; Lamirault & Simon, 2001; Marchetti et al., 2011). These effects are blocked by 5-HT_4_R antagonists, indicating a direct receptor-mediated mechanism (Fontana et al., 1997). There are a number of potential mechanisms of these pro-cognitive effects, including enhanced hippocampal synaptic plasticity (Kozono, Ohtani, & Shiga, 2017), increased acetylcholine release (Siniscalchi, Badini, Beani, & Bianchi, 1999), and modulation of glutamate transmission (Chen et al., 2020).

Early translational studies in healthy volunteers (de Cates et al., 2021; de Cates et al., 2022; Murphy, Wright, Browning, Cowen, & Harmer, 2020) have demonstrated that the selective 5-HT_4_R agonist prucalopride has a pro-cognitive profile in humans. In these studies, acute and subchronic (6 days) administration of prucalopride has been shown to improve learning and memory across a range of cognitive tasks, including predominately ‘cold’ non-emotionally valenced cognitive tasks involving declarative memory and reward learning. Prucalopride also improves the accuracy of task performance in paradigms without a memory component, which may be indicative of enhanced attentional function. For example, prucalopride has been shown to increase the accuracy with which healthy volunteers are able to identify the gender of rapidly presented faces (de Cates et al., 2022). Consistent with these pro-cognitive behavioral effects, neuroimaging data show that prucalopride increases bilateral hippocampal during a memory task (de Cates et al., 2021) and decreases default mode network (DMN) functional connectivity (de Cates et al., 2023). In contrast, prucalopride does not show significant effects on ‘hot’ emotional processing that mirror those seen with conventional antidepressants in healthy volunteers (Lucas et al., 2005; Lucas et al., 2007; Mendez-David et al., 2014).

Together, this evidence positions 5-HT_4_R agonism as a promising approach for treating cognitive dysfunction in depression and potentially preventing future depressive episodes. Furthermore, recruiting individuals with remitted depression (but who are currently not meeting criteria for acute depression) enables the assessment of the effects of the intervention on cognition while minimizing the potential confounds of acute mood symptoms and concurrent medication (Colwell et al., 2022). The current study aimed to investigate the cognitive effects of prucalopride in individuals with a history of recurrent depression, currently not meeting ICD-10 criteria for depression for at least 6 months and free of psychotropic medication. The study extends previous work by testing a higher dose of prucalopride (2 mg) and by including a broader battery of cognitive tests to further characterize the effects of 5-HT_4_R agonism on attention and executive function, as well as memory. We hypothesized that prucalopride would exert a broad pro-cognitive effect in this population: with a greater effect on ‘cold’ cognitive tasks as opposed to those involving emotional cognition.

## Methods

### Participants

Participants between 18 and 40 years old and of either sex were recruited to the study. They were screened to exclude those with contraindications to serotonergic medication. All participants were currently free of significant medical or psychiatric disorder but had a history of at least two episodes meeting DSM-5 criteria for major depressive disorder, as determined using the Structured Clinical Interview for DSM-5 (SCID). Other criteria for inclusion were as follows: English language fluency, at least 6 months recovery since most recent depressive episode, current PHQ-9 score of less than 10, no current use of any medication except contraception, and no medical concerns related to the use of prucalopride. Participants were excluded if they met criteria for any current axis 1 DSM-5 psychiatric disorder, had a first-degree relative with bipolar affective disorder type 1/schizophrenia, or had a significant medical condition that might cause safety concerns for the study (e.g. inflammatory bowel disease). Full exclusion criteria are detailed in Supplementary Table S1.

The study was approved by the University of Oxford Central University Research Ethics Committee (CUREC, R77135/RE005). The protocol was pre-registered with clinicaltrials.gov (NCT05220228). Participants were recruited via adverts emailed to and displayed in colleges and university departments and local community buildings, placed on local information websites, newspapers, local magazines, and on the lab webpage. We also recruited participants using social media advertisements, supported by Lindus Health (a clinical research recruitment service). Participants gave written informed consent. Full details of all methods are in the Supplementary Material.

### Design and randomization

#### Overview of design and randomization

Participants were randomized to one of two groups: prucalopride (2 days × 1 mg followed by 5–8 days × 2 mg) or placebo (lactose tablets for 7–10 days) in a double-blind, randomized, between-groups design. Randomization was stratified by gender and was conducted using an online randomization tool with block size of 4 (https://www.sealedenvelope.com/simple-randomiser/v1/lists). Prucalopride and placebo tablets were encapsulated using a standardized operating procedure to ensure that they appeared identical. Participants received two bottles to take consecutively: bottle one had two capsules of 1 mg prucalopride or placebo (for first 2 days of administration); bottle two had eight capsules of 2 mg prucalopride or placebo. The full licensed dose of prucalopride (2 mg) was used for at least 5 days to achieve steady state (terminal half-life approximately 24 h). Daily reminders optimized compliance.

Our previous translational studies of prucalopride in healthy volunteers employed a daily dose of 1 mg which was well-tolerated. However, 2 mg daily is the licensed dose for prucalopride and uncertainty exists regarding the dose required for full effect at brain 5-HT_4_ receptors. As previous studies in healthy volunteers suggested that a single dose of prucalopride has pro-cognitive effects on declarative memory with effect sizes ranging from *d* = 0.5–0.9 (Murphy et al., 2020), in this study (with a higher dose and longer duration), we conservatively estimated *d* = 0.5–0.7, and thus we calculated 23 participants per group would be needed to achieve power of 85–90% and alpha of 0.05.

#### Study visits

Two study visits (baseline and follow-up testing after at least 7 days of prucalopride/placebo) took place at the Neurosciences Building, Department of Psychiatry, Warneford Hospital following initial screening. At the point of post-intervention testing, prucalopride blood levels at 2 mg daily would be expected to be at a steady state.

### Questionnaire measures

Participants completed the following self-report questionnaires to obtain baseline measures of mood, affect, cognitive difficulties, anhedonia, and trait anxiety: PHQ-9, Positive and Negative Affect Scale (PANAS; Watson, Clark, & Tellegen, 1988), Perceived Deficits Questionnaire (PDQ20; Sullivan, Edgley, & Dehoux, 1990), Snaith–Hamilton Pleasure Scale (SHAPS; Snaith et al., 1995), and Spielberger State–Trait Anxiety Inventory, Trait Version (STAI-T; Spielberger, Gorssuch, Lushene, Vagg, & Jacobs, 1983). Post-intervention, at the start of the research testing visit, participants’ state anxiety and affect were measured using the Spielberger State–Trait Anxiety Inventory, State Version (STAI-S; Spielberger et al., 1983) and the PANAS. Binary data regarding the most commonly reported side effects of prucalopride (Frampton, 2009) were measured both pre- and post-intervention to give a measure of side effects across the course of the study. At the end of the study, participants guessed their drug allocation with a forced-choice question.

### Cognitive tasks

We evaluated participant performance across a range of ‘cold’ cognitive tasks that did not contain emotionally valenced stimuli (the auditory verbal learning and memory task testing declarative memory [AVLT], a working memory task [N-back], and tests of executive functioning [including attention and processing speed: TMT, DSST]). We also used ‘hot’ cognitive tasks that included a component of processing relating to emotionally valenced information. These included the emotional go/no-go (E-GNG) task, the emotional test battery (ETB), and the facial attentional dot probe task (FDOT). Multiple versions were available for the AVLT, N-back, DSST, and E-GNG, enabling data collection at baseline and post-intervention; other tasks were conducted post-intervention only. Apart from for the AVLT (auditory stimuli and verbal responses) and DSST and TMT (paper-based), all task stimuli were presented on a computer screen and participants required to respond via button presses on a keyboard. Full details regarding all tasks are given in Supplementary Material, and each is outlined briefly here. Non-emotional tasks were pre-determined as primary and emotional tasks as secondary for analysis purposes (see clinicaltrials.gov NCT05220228).

#### ‘Cold’ cognitive tasks

**Figure 1.**
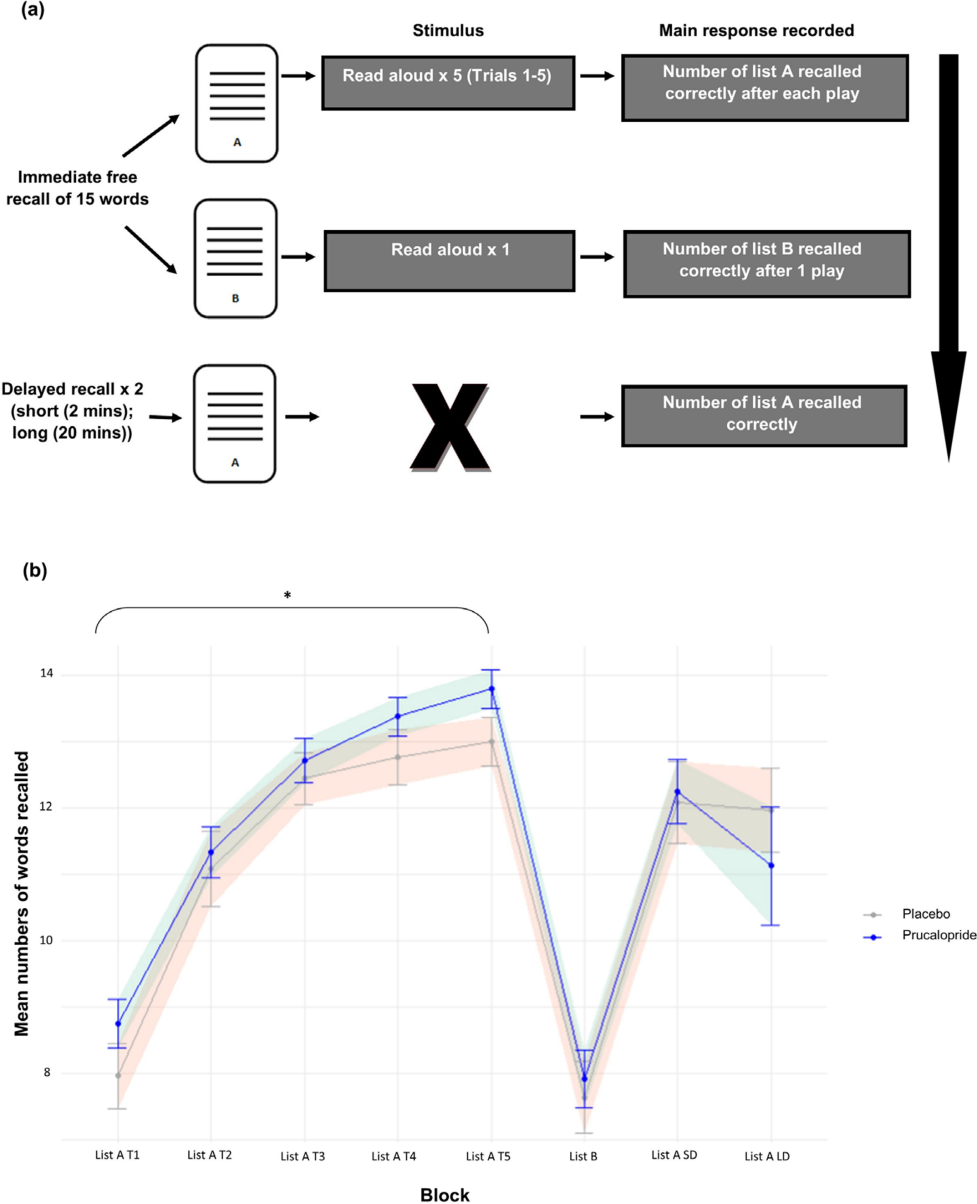
Mean number of words recalled across the auditory verbal learning task (AVLT) comparing the prucalopride and placebo groups. (a) Participants were read 15 concrete nouns from List A at a rate of one word per second. Participants were asked to immediately verbally recall as many items as they could, in any order. This was repeated a further four times, comprising five acquisition trials. Participants were then read a second set of 15 nouns (List B) and asked to recall words from this second list only. Immediately following List B, participants were asked to recall List A (short-delay), and once more after a delay of approximately 20 minutes, during which another task was completed (long-delay). (b) Error bars and shaded area indicate the standard error of the mean. * represents statistical significance at *p* = 0.05. Graphs with baseline data included are in Supplementary Material. Figure 1. long description.

*Auditory verbal learning task*. In the auditory verbal learning task (AVLT) (see Figure 1a), participants’ verbal learning and memory was assessed using a multi-trial word recall paradigm. Fifteen concrete nouns (List A) were read aloud across five consecutive learning trials. After each presentation, participants were asked to immediately recall as many words as possible. Following the fifth trial, an interference list comprising 15 unrelated words (List B) was presented, with immediate recall subsequently assessed. Participants were then asked to recall the original List A words after a short delay (short-delay recall) and again following a longer delay of approximately 20 minutes (long-delay recall). Outcome measures included the total number of correct words recalled, repetitions (correct words recalled more than once in the same acquisition trial), and intrusions (incorrect words not present in the list).

**Figure 2.**
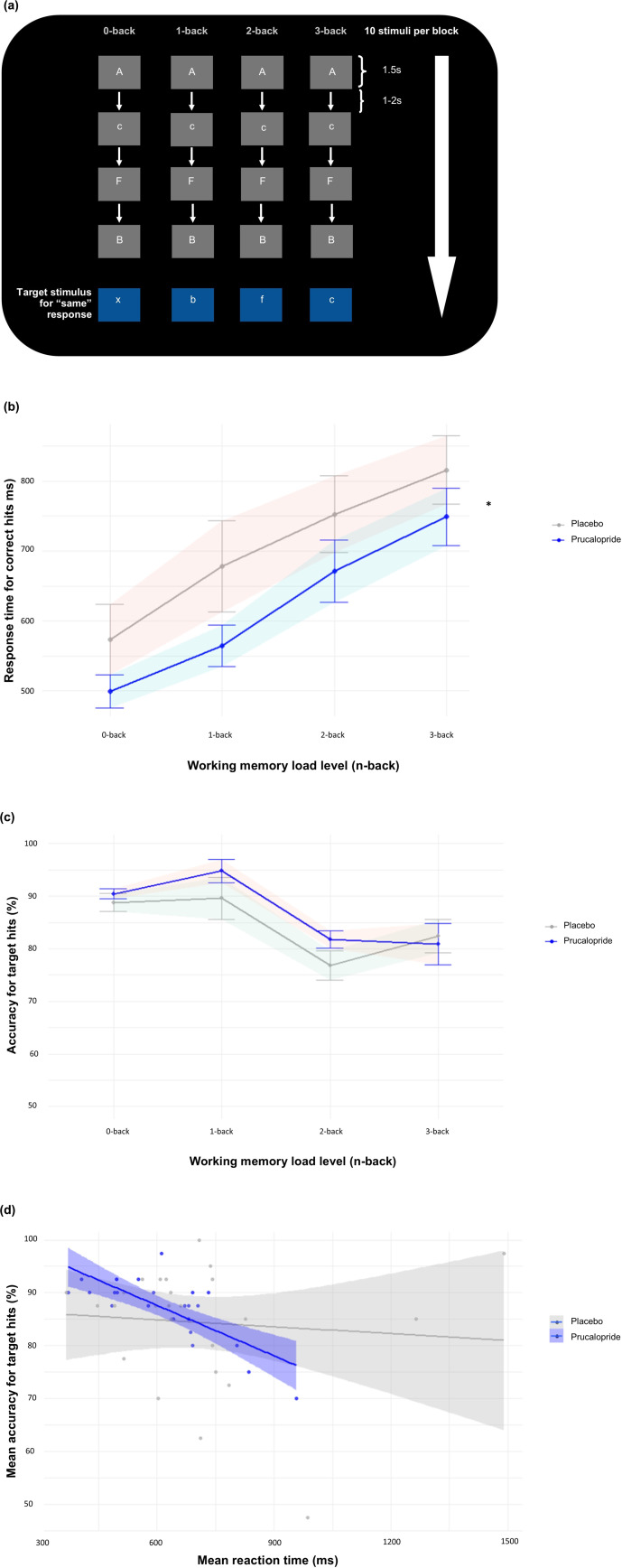
Results for the N-back task according to working memory load in the prucalopride and placebo group. (a) Verbal n-back task exemplar for conditions of 0-back, 1-back, 2-back, and 3-back. Before each block of 10 stimuli per condition, participants were given specific instructions (e.g. ‘Press the spacebar if you see the same letter that appeared 1 letter ago’ [1-back]). Each level of back was repeated four times (16 blocks total). For 0-back, participants were asked to select ‘same’ only when the letter ‘X’ appeared. For 1-back/2-back/3-back, participants were asked to select ‘same’ if the current letter was the same as the one/two/three before. (b) Mean response time (ms) for correct hits; (c) mean accuracy (%) for correct hits; and (d) mean accuracy compared to mean response time for correct hits. Error bars indicate the standard error of the mean. The shaded area indicates 95% confidence interval. * represents statistical significance at *p* = 0.05. Figure 2. long description.

*N-back.* In the N-back task (see Figure 2a), participants were required to indicate whether a visually presented symbol matched the one presented ‘n’ trials earlier, where ‘n’ corresponded to zero (0-back), one (1-back), two (2-back), or three (3-back). This task incrementally increased working memory demands across conditions. Performance was assessed based on reaction time and accuracy at each level of difficulty.

*Digit-symbol substitution task*. In the digit-symbol substitution task (DSST), participants were required to match symbols to corresponding letters using a reference key. Performance was evaluated by recording the total number of correct symbol entries and the time taken to complete the task, providing a measure of processing speed and cognitive efficiency.

*Trail-making task*. The trail-making task (TMT) assessed processing speed, attention, and cognitive flexibility. It consisted of two parts: Part A required participants to connect consecutively numbered circles (e.g. 1-2-3), while Part B involved connecting circles in an alternating sequence of numbers and letters (e.g. 1-A-2-B). Performance was evaluated based on completion time and accuracy for each part. Additionally, the difference in completion time between Part B and Part A (B-A) was calculated as an index of executive functioning, specifically set shifting ability.

*Composite analysis*. We performed an additional composite analysis, focusing on ‘cold’ cognition tasks, which we specified *a priori* as the primary cognitive domain of interest. This included ‘cold’ cognitive tasks with pre- and post-intervention data to assess mean change across tasks (as a z score) between these timepoints compared across our groups (prucalopride versus placebo). For reaction time analyses, this comprised the DSST and N-back, and for performance analyses, this included the AVLT and N-back. Mean change for each task for each group was calculated using the difference between post- and pre-intervention scores or reaction times and converted into a z score. Z scores were then averaged across tasks within each group for accuracy and reaction time, respectively, and compared at the group level. Consistent with conventional benchmarks for standardized effect sizes, composite z-scores with absolute magnitude greater than 0.5 were interpreted as reflecting at least a moderate effect.

#### ‘Hot’ cognitive tasks

**Figure 3.**
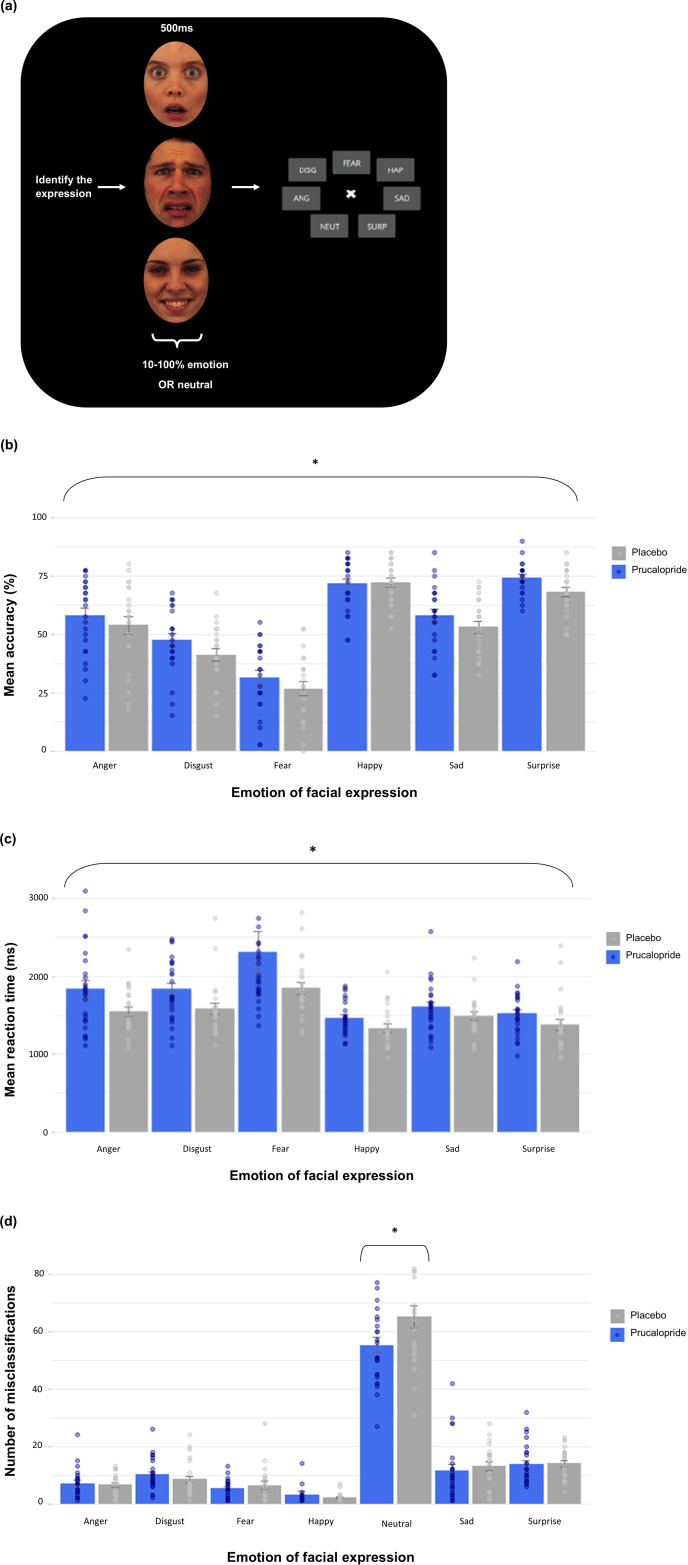
Mean accuracy and response time for correct responses and misclassifications on the facial expression recognition task (FERT) showing prucalopride and placebo group responses. (a) 250 randomized face images (emotions [anger, disgust, fear, sadness, surprise, and happiness] and neutral) were shown for 500 ms in varying intensities in 10% gradations ranging from 0% (neutral) to 100% (full emotion). Participants were asked to identify the emotion presented. (b) Mean accuracy (%); and (c) mean reaction time (ms); and (d) mean number of misclassifications. Error bars indicate the standard error of the mean. * represents statistical significance at *p* = 0.05. Figure 3. long description.

*Emotional test battery*. The emotional test battery (ETB) included four tasks assessing emotional processing. In the facial expression recognition task (FERT) (see Figure 3a), participants identified facial expressions (angry, disgusted, fearful, happy, sad, and surprised) presented briefly (500 ms) on a computer screen. Expressions were displayed at varying intensities, ranging from neutral to 100% full emotion in 10% increments. Accuracy to identify the correct emotion, reaction time, and misclassification rates were recorded. In the emotional categorization task (ECAT), participants indicated whether they would like or dislike being described as positive and negative personality descriptor words. Classification accuracy and reaction times were measured. In the emotional recall task (EREC), participants completed an unexpected free recall of the words previously presented in the ECAT, with the number of correctly recalled positive and negative words, as well as false alarms, recorded. Finally, in the emotional recognition task (EMEM), participants identified whether a series of words presented on the screen were familiar (previously seen in the ECAT) or novel. Accuracy and response time were measured.

*Faces dot probe task*. The faces dot probe task (FDOT) measures attentional vigilance to fear and happy faces, as previously described (Murphy, Yiend, Lester, Cowen, & Harmer, 2009). Participants were shown pairs of faces – one emotional (fearful or happy) and one neutral – positioned at the top and bottom of the screen. These were then replaced by a pair of dots (probe) aligned either vertically or horizontally. Participants were instructed to indicate the orientation of the dots by pressing a labeled key. On half of the trials (unmasked condition), the face pair was presented for 100 ms and immediately followed by the probe. On the other half of the trials (masked condition), the sequence of events was exactly the same except the face pair was only presented very briefly (16 ms) and followed by a mask (constructed from a jumbled face), which was displayed for 84 ms. Attentional vigilance toward the emotional stimuli was assessed by calculating vigilance scores. These were derived by subtracting reaction times on incongruent trials (i.e. where the probe appeared in the location of the neutral expression) from congruent trials (i.e. where the probe appeared in the location of the emotional face), after reaction times for incorrect trials were removed. Positive vigilance scores indicated a bias toward emotional stimuli.

*Emotional go/no-go task*. This task measured behavioral inhibition in the presences of affective interference, using happy, fearful, and control (neutral/scrambled) images as distractors, as previously described (Colwell et al., 2024). Participants were instructed to respond (Go) or withhold responses (No-Go) based on shifting task rules (e.g. ‘Do not press the button if the image is yellow’) while simultaneously being presented with an emotional distractors (a fearful or happy face) or a control image. These task rules shifted across blocks. Emotional faces were irrelevant to the task rules but served as distractors to introduce emotional interference. Outcome measures included (1) accuracy on No-Go trials, reflecting response inhibition; (2) accuracy on Go trials; and (3) response times on Go trials, providing an index of impulsivity and processing speed.

### Data analysis

Behavioral data and questionnaires were analyzed and graphs produced using RStudio (version 4.3.3). Two-sided *t*-tests, chi-squared tests, and analysis of variance (ANOVA) were used as appropriate for demographic and questionnaire analyses. Levene’s test (*t*-tests) and the Greenhouse–Geisser procedure (ANCOVAs/ANOVAs) were used as required. A repeated-measures analysis of co-variance (ANCOVA) was used as pre-specified to analyze group differences in behavioral performance on tasks suitable for repeated testing, with baseline data for the task used as the covariate (AVLT, N-back, DSST, EGNG) and estimated marginal means for planned comparisons after ANOVAs. For the AVLT, mean of list A scores across the first five blocks at baseline and task version order were included as covariates. For the emotional go/no-go (EGNG), we also conducted drift diffusion modeling. A *p*-value less than 0.05 was used to denote statistical significance. Partial eta squared (np2) and Cohen’s *d* are reported as appropriate as measures of effect size. Sensitivity analyses involved the following included as covariates: baseline mood (PHQ9) and subjective cognition scores (PDQ20) (all tasks), age and years of education (executive functioning tasks [DSST, TMT]), and native language (verbal processing tasks [EREC, ECAT, EMEM]). All sensitivity analyses are in Supplementary Material and are reported where they differ from main analyses.

## Results

### Participants

In total, 50 participants were randomized into the study across two groups (placebo and prucalopride groups [25:25]) between 3rd March 2022 and 23rd October 2023 (see Supplementary Figure S1). Seven additional participants were recruited but replaced due to active Covid infection during the study or ineligibility determined after randomization. One participant (prucalopride group) was excluded prior to unblinding for data quality issues, resulting in 49 participants for analysis (25:24; placebo:prucalopride). Analysis occurred in originally assigned groups. Data were not collected for the emotional test battery tasks for one participant allocated to the prucalopride group as they had recently undertaken this during another study. Participants received the intervention for a similar period across groups [mean (SD): placebo 7.08 (0.28) days, prucalopride 7.25 (0.61) days].

At baseline, the groups were well matched in terms of demographics (i.e. age, sex, BMI, ethnicity, first language, years of education, handedness, and substance use; see Table 1), as well as severity of subjective cognitive problems (PDQ; Sullivan et al., 1990), affect (PANAS; Watson et al., 1988), and trait anxiety (STAI-T; Spielberger et al., 1983). Mean PHQ-9 score at baseline indicated low depressive symptomatology (consistent with remitted depression) (mean [SD]: placebo 3.08 [2.71]; prucalopride 2.38 [2.24]) and was similar across the two groups.

**Table 1.** Baseline demographics, mood, and affect symptoms for the progress study Table 1. long description.

Variable (measure)	Measurement		Placebo*N* = 25	Prucalopride*N* = 24
*Sex*	*%*	*Male*	36.0	29.0
		*Female*	64.0	71.0
*Age*	*Mean in years (SD; range)*		30.2 (9.95)	28.0 (7.16)
*Years of education*	*Mean (SD)*		17.6 (3.04)	17.8 (2.63)
*First language*	*%*	*English*	72.0	91.7
		*Not English*	28.0	8.3
*Ethnicity*	*%*	*White*	56	50
		*Asian*	28	16.7
		*Black*	4	8.3
		*Other/mixed*	12	25
*Current smokers*	*%*		12.0	16.7
*Alcohol*	*Mean units/week (SD)*		2.53 (3.43)	2.66 (3.39)
*Caffeine*	*Mean cups/day (SD)*		1.53 (1.35)	1.30 (1.10)
*Body mass index*	*Mean (SD)*		24.7 (3.63)	23.4 (3.25)
*Systemic contraception*	*%*		28	13
*Depression* *PHQ-9*	*Mean (SD)*		3.08 (2.71)	2.38 (2.24)
*Subjective cognition (PDQ-20)*	*Mean (SD)*		25.3 (11.8)	19.4 (11.7)
*Anhedonia (SHAPS)*	*Mean (SD)*		0.60 (1.63)	0.50 (0.83)
*Anxiety (STAI-T)*	*Mean (SD)*		33.6 (8.7)	38.0 (10.1)
*Affect (PANAS)*	*Mean (SD)*	*Positive*	31.3 (6.57)	33.8 (7.62)
		*Negative*	12.7 (3.12)	11.8 (2.26)

### Tolerability of prucalopride during the interventional period

Prucalopride was well tolerated compared to placebo administration. Binary ratings of side effects (present/non-present during the last week) were analyzed using two-way ANOVAs with time point (pre-intervention, post-intervention) and group as factors. There were no significant time × group interaction differences in reports of total side effects [F(1,47) = 0.12, p = 0.73, np^2^ < 0.01], or headache, abdominal pain, nausea, diarrhea, dizziness, vomiting, flatulence, or gastrointestinal sounds when analyzed individually (all ps > 0.3, see Supplementary Table S2). There was a group × time interaction for the decreased appetite item, which reflected significantly higher scores in the prucalopride group post-intervention [F(147) = 5.42, *p* = 0.024; placebo participants endorsing at baseline = 12% and follow-up = 4%; prucalopride participants endorsing at baseline = 12% and follow-up = 24%]. Further detail is in Supplementary Material.

Randomization guesses (data missing for one placebo participant) suggested that most placebo participants guessed their allocation correctly, whereas prucalopride participants were at around chance levels [correct guess: placebo 83.0% (20/24), prucalopride 58.3% (14/24); χ^2^ = 4.61, *p* = 0.10].

### Subjective anxiety, affect, and mood

There were no significant differences between the two groups in state anxiety and negative affect at the start of the post-intervention research visit [STAIS-S (mean (SD): placebo 30.7 (6.65), prucalopride 31.7 (9.90); *t*(40.1) = −0.37, *p* = 0.71); PANAS-N (mean (SD): placebo: 12.32 (2.93), prucalopride 12.5 (4.11); *t*(41.4) = −0.18, *p* = 0.86)]. However, in the prucalopride group, there was a borderline significant difference between the groups in terms of positive affect at the post-intervention research visit, with higher scores in the prucalopride group compared with the placebo group [PANAS-P mean (SD): placebo 29.1 (6.98), prucalopride 33.2 (7.35), *t*(46.6) = −1.99, *p* = 0.05, Cohen’s *d* = −0.57 (−1.14, 0.00)], which was not wholly explained by PANAS scores at baseline (see Table 1)].

Subjective mood was not significantly different post-intervention compared to baseline across groups [mean (SD): baseline PHQ9 placebo 3.08 (2.71) prucalopride 2.38 (2.24); post-intervention QIDS-C placebo 4.16 (2.61) prucalopride 3.58 (2.50); *F*(1,94) = 1.67, *p* = 0.20]; see Supplementary Figure S2 using equivalence tables from Palmer, Ker, Rentería, Carmody, & Rush (2024).

### Does 5-HT_4_ receptor agonism affect non-emotional ‘cold’ cognition in those with remitted depression?

#### Declarative memory (Auditory verbal learning task (AVLT): placebo 25, prucalopride 24)

In both groups, participants’ recall of words from List A improved across the five acquisition blocks (see Figure 1b and Supplementary Figure S3A,B). Participants in the prucalopride group were on average more accurate than those in the placebo group in their recall across these five acquisition blocks [*F*(1,226) = 8.12, *p* = 0.005, np2 = 0.16]. There was no statistically significant group × block interaction [*F*(4,226) = 0.45, *p* = 0.77], indicating that recall was improved on average across all the task blocks.

There was no difference between the prucalopride and placebo participants in terms of the number of words accurately recalled after a short delay [*F*(1,42) = 0.016, *p* = 0.90] or a long delay [*F*(1,42) = 0.96, *p* = 0.33], nor for intrusions, repetitions, or recall of a separate set of words (List B) (all *p*s > 0.3, see Supplementary Figure S3C).

#### Verbal working memory (N-back: placebo 25, prucalopride 23)

The prucalopride group were significantly quicker on average across task loads (0- to 3-back) [*F*(1,179) = 8.56, *p* = 0.004, np2 = 0.06, see Figure 2b]. There was no group × condition interaction (*F*(3,179) = 1.51, *p* = 0.21. The prucalopride group tended to make correct choices more quickly although the group × reaction time interaction fell short of significance [group × mean reaction time: *F*(1,44) = 3.17, *p* = 0.082]; see Figure 2d and Supplementary Table S5. This was also reflected by the relationship between d’ and group (*t*(−1.68, 45.53), *p* = 0.099, see Supplementary Figure S4). Findings were unchanged in sensitivity analyses, although when we excluded a placebo participant who was an outlier for reaction time (reduced speed) but not for accuracy the main effect of group for reaction time became borderline [*F*(1,175) = 3.71, *p* = 0.05, np2 = 0.01].

There was no main effect of group in terms of accuracy for recalled targets [*F*(1,179) = 2.12, *p* = 0.15,), although there was a trend for increased accuracy at the 1- and 2-back level (see Figure 2c; 0-back EMM: −0.16 ± 0.40, *p* = 0.68, *d* = −0.12 [−0.69–0.45]; 1-back EMM: −0.52 ± 0.40, *p* = 0.19, *d* = −0.38 [−0.95–0.19]; 2-back EMM: −0.49 ± 0.40, *p* = 0.21, *d* = −0.36 [−0.93–0.21]; 3-back EMM: 0.15 ± 0.40, *p* = 0.70, *d* = 0.11 [−0.46–0.68]). Data from one participant (prucalopride) were missing for the N-back.

#### Tasks assessing executive function: (DSST: placebo 25, prucalopride 24; TMT: placebo 24, prucalopride 22)

There were no statistically significant group effects for performance for the digit-symbol substitution task [*F*(1,45) = 0.77, *p* = 0.38] or the trail-making task (TMTA: [*t*(44.0) = 0.007, *p* = 0.99]; TMTB-TMTA: [*t*(37.1) = 0.058, *p* = 0.56]).

#### Composite analysis across ‘cold’ cognitive tasks

Prucalopride administration was associated with increased accuracy (*z* = +0.59: AVLT; N-back) and faster response times (*z* = −0.69: N-back; DSST) when compared to the placebo group.

### Is the processing of emotions (‘hot’ cognition) affected by 5-HT_4_ receptor agonism in previous depression?

#### Facial expression recognition task (FERT: placebo 25, prucalopride 23)

Participants in the prucalopride group were more accurate than placebo participants across all emotions [*F*(1,270) = 9.99, *p* < 0.002, np2 = 0.05]. However, there was no interaction between group and emotion [*F*(5,270) = 0.44, *p* = 0.82]; see Figure 3b. Participants in the prucalopride group were also significantly slower than those in the placebo group across all emotions [*F*(1,269) = 13.5, *p* < 0.001, np2 = 0.13], with no group × emotion interaction [*F*(5,269) = 0.65, *p* = 0.66]; see Figure 3c.

Consistent with the overall effect of group on accuracy, the prucalopride group made fewer misclassifications of other faces as neutral [*F*(1,42) = 5.59, *p* = 0.02, np2 = 0.10]; see Figure 3d. Inverse efficiency score analyses did not demonstrate a significant effect for allocation (*F*(1,314) = 0.68, *p* = 0.41) or allocation × emotion (*F*(6,314) = 0.53*, p* = 0.79).

#### Faces dot probe task (FDOT: placebo 24, prucalopride 22)

The prucalopride group was significantly slower than the placebo participants across all trials [*F*(1,733) = 9.06, *p* = 0.0027, np2 = 0.01, placebo mean (SD) 0.55 s (0.08), prucalopride mean (SD) 0.57 s (0.06), see Supplementary Figure S7A]. There was no statistically significant group level effect in terms of attentional vigilance [*F*(1,686) = 0.28, *p* = 0.60, see Supplementary Figure S7B]. There was a group × mask × emotion interaction [*F*(1,686) = 4.19, *p* = 0.04, np2 = 0.006]; however, this was no longer significant when examining group × emotion interactions for the masked and unmasked conditions separately [masked: *F*(1,344) = 2.42, *p* = 0.12; unmasked: *F*(1,342) = 1.81, *p* = 0.18]; see Supplementary Figure S7C and Supplementary Table S11. Results were unchanged when we calculated proportional difference scores instead of vigilance to take account of the group-level difference in reaction time (see Supplementary Material).

#### Emotional go/no-go task (placebo 25, prucalopride 24)

5-HT_4_R agonism was not associated with a change in response inhibition (measured by mean percentage of accurately withheld responses to no-go trials) (ANCOVA main effect of group: *F*[1,46] = 0.065, *p* = 0.799; placebo mean (SD) 34.8 (38.4); prucalopride mean (SD) 34.4 (37.9); all conditions EMM = 0.31 ± 1.84, *p* = 0.87 (Supplementary Figure S8A), a group by set shift interaction for accuracy of withheld presses (ANCOVA: *F*[1,340] = 0.107, *p* = 0.74) or a group effect for go trial accuracy (ANCOVA main effect of group: *F*[1,46] = 0.004, *p* = 0.95) when considering either the control condition or affective interference (Supplementary Figure S8B).

Prucalopride allocation also did not affect choice impulsivity, indicated by no change in reaction time to choice for go trials, across all task conditions (ANCOVA main effect of group: *F*[1,46] = 0.23, *p* = 0.63) (see Supplementary Figure S8C).

Signal detection theory analyses suggested that prucalopride allocation did not influence decision bias across task conditions (log criterion c; ANCOVA main effect of group: *F*[1,46] = 0.96, *p* = 0.33; all conditions EMM = −0.015 ± 0.03, *p* = 0.58) (see Supplementary Figure S8D); no other significant differences were evident between groups in drift diffusion modeling (see Supplementary Table S10 and Supplementary Figure S8E).

Controlling for baseline mood and subjective cognition did not affect results (see Supplementary Material). When we excluded two participants (1 prucalopride, 1 placebo) each with trials where reaction times were outside optimization parameters (i.e. too short indicating lack of engagement), results were similar apart from log criterion analyses (log criterion c), which became significant (see Supplementary Material).

#### Emotional test battery (placebo 25, prucalopride 23)

There were no statistically significant group effects for performance or reaction time on the emotional categorization task (ECAT), the emotional recall task (EREC), and the emotional recognition memory task (EMEM): all *ps* > 0.3. This included when sensitivity analyses were performed controlling for baseline mood and subjective cognition scores (see Supplementary Material).

## Discussion

In this experimental medicine study of participants with a history of depression, 7–10 days of prucalopride administration, compared to placebo, were associated with improved performance on several cognitive measures. Specifically, prucalopride improved immediate recall on an auditory verbal memory task (AVLT), increased accuracy in facial expression recognition (FERT) regardless of emotional valence, and led to faster responses on a complex working memory task (N-back) with a trend toward improved accuracy. To mitigate the risk of Type I error while preserving sensitivity to domain-level effects, and in line with our pre-registration on clinicaltrials.gov, we performed an additional composite analysis, focusing on ‘cold’ cognition tasks (which we specified *a priori* as the primary cognitive domain of interest). This approach provides a robust and theoretically meaningful index of cognitive change that complements and strengthens the individual task-level tests. This composite analysis of ‘cold’ cognitive tasks identified that prucalopride administration was associated with faster responses and increased accuracy compared to participants receiving placebo.

These observed pro-cognitive effects of prucalopride (2 mg) are consistent with previous translational work in healthy volunteers using a 1-mg dose. In earlier studies, prucalopride improved multiple cognitive domains, including declarative memory, reward learning, emotional memory, and face processing (de Cates et al., 2021; Murphy et al., 2020). Our findings extend these effects to a remitted depressed sample and replicate improvement in immediate recall. Importantly, we demonstrate additional benefits with a broader profile of pro-cognitive effects, including on tasks of working memory.

On the N-back task, the prucalopride group responded faster across difficulty levels, with a trend toward improved accuracy. This contrasts with previous studies using a single 1-mg dose, which did not detect significant effects of prucalopride on working memory (Murphy et al., 2020). In the current study, participants reached steady-state dosing (≥ 5 days) of 2 mg, potentially enhancing observable effects in this cognitive domain. Our findings are also consistent with other work showing that 5-HT_4_R agonism is associated with faster reaction times without accuracy impairments in people with depression (de Cates et al., 2025).

Interestingly, reaction time patterns varied across tasks. Compared to placebo participants, the prucalopride group was slower on tasks involving emotional processing (FERT, FDOT), potentially reflecting more cautious and accurate responding. Conversely, as discussed, the prucalopride group was significantly faster on the N-back task, which reflects non-affective working memory.

There was also limited evidence that 5-HT_4_R agonism influenced emotionally valenced cognitive performance; improvements on the FERT were not emotion-specific, and no significant effects on accuracy were observed for the emotional go/no-go, FDOT, ECAT, EREC, or EMEM tasks. These limited effects of prucalopride on emotional cognition are consistent with previous work (de Cates et al., 2022) and with another recent 5-HT_4_R study from our groups (unpublished data). In this study, participants with current depression showed mood improvements following sub-acute administration with both an unlicensed 5-HT_4_R agonist (PF-04995274) and citalopram but only citalopram altered emotional processing biases – a key mechanism through which conventional antidepressants exert their effects (Harmer, Goodwin, & Cowen, 2009). This suggests that prucalopride’s mechanisms may be distinct from emotional bias modification.

In this study, we replicated our previous findings in healthy volunteers demonstrating pro-cognitive effects following a single acute dose of prucalopride (Murphy et al., 2020). This is consistent with preclinical evidence demonstrating that 5-HT4 receptor agonists produce a rapid increase in levels of BDNF after only a few doses, in contrast to selective serotonin reuptake inhibitors, which typically require several weeks of administration to produce similar BDNF upregulation (Lucas et al., 2007). Preclinical studies further suggest that these behavioral and neurochemical changes may be sustained for 14–30 days (Hashemi-Firouzi, Shahidi, & Soleimani Asl, 2021; Quiedeville et al., 2015); however, the longer-term effects of 5-HT_4_ receptor agonism are yet to be examined in humans. Whether procognitive effects of prucalopride are further enhanced with sustained administration, or if they persist after cessation of treatment, remain important questions to be explored in future research.

Several neurobiological mechanisms may underlie the pro-cognitive effects of 5-HT_4_R agonism. First, 5-HT_4_Rs are expressed in the hippocampus, as well other regions of the temporal lobe, parietal lobe, and densely expressed in the basal ganglia (Beliveau et al., 2017). Rodent studies show improvements in hippocampal-dependent learning and memory following 5-HT4R activation (Lamirault & Simon, 2001; Marchetti et al., 2011; Mohler et al., 2007; Pfizer, 2011). Potential mechanisms include modulation of AMPA receptor-mediated glutamatergic transmission (promoting stress resilience) (Chen et al., 2020), increased acetylcholine release (Hagena & Manahan-Vaughan, 2017), and enhanced neurogenesis and dendritic growth (Lucas et al., 2007). Given the known hippocampal abnormalities in depression – including reduced hippocampal volume and disrupted functional connectivity with the prefrontal cortex and amygdala (Cao et al., 2012; Hao et al., 2020; Sheline, Liston, & McEwen, 2019); (Cullen et al., 2014; Hamilton & Gotlib, 2008) – these mechanisms may have particular clinical relevance. Furthermore, the significant effects of prucalopride on performance of the AVLT and the N-back are consistent with facilitated function of the hippocampus. Second, 5-HT_4_R agonism may exert effects on cognition and mood via the gut-brain axis. The vast majority of serotonin is produced in the gut, and peripheral serotonergic signaling may influence central cognitive flexibility (Shine et al., 2022). Notably, antagonism of 5-HT_4_Rs has been linked to pro-inflammatory microbiome profile, suggesting that these receptors may also regulate gut–brain inflammation (Cui et al., 2023).

Given the prevalence of persistent cognitive problems after remission of depressive illness (Rock et al., 2014), and their association with relapse risk (Halahakoon et al., 2019), interventions that target and improve cognition could offer meaningful clinical benefits. Our findings were unchanged by including mood or subjective cognition scores at baseline into analytical models, suggesting that the potential pro-cognitive impact of 5-HT_4_R agonists may be relevant across mood states, including for individuals with subclinical or unrecognized cognitive impairments.

There are some limitations of the current study that should be considered. The study was underpowered for some tasks due to missing data (e.g. TMT sample was reduced from n = 24 to n = 22 in the prucalopride group). As the purpose of our study was primarily mechanistic, and as is typical for experimental medicine studies, sample sizes were powered (*n* = 23 per group) to explore mechanisms and proxy measures of effect using a population sample with similar characteristics. This enables rapid recruitment and dissemination of exploratory findings to provide evidence for further studies evaluating efficacy where diverse populations are recruited. Although our sample included participants with varied histories of antidepressant use and number of previous episodes – supporting generalizability – it was predominately female, white and highly educated, and limited to adults under the age of 40 (to reduce variability in cognitive function including processing speed) (Hughes, Agrigoroaei, Jeon, Bruzzese, & Lachman, 2018). Most participants (<30%) were not receiving systemic hormonal contraception across the groups (28% prucalopride: 13% placebo; see Table 1). Limiting variability within study populations can support power in smaller mechanistic studies but may restrict the applicability of findings to broader, more diverse populations. For example, the study was not powered to examine whether biological sex or contraception use moderated the effects of prucalopride on cognition. However, this may be important to examine in future studies as there is evidence that 5-HT_4_R binding may be differentially mediated according to sex and hormonal contraception (Jensen et al., 2025).

Furthermore, cognitive deficits were not part of study inclusion criteria, raising the possibility that some participants may have had limited baseline cognitive impairment. However, baseline PDQ scores indicated the presence of mild to moderate subjective deficits in cognition consistent with what might be expected for this remitted depressed population. Notably, we observed improvements in cognitive performance and reaction times despite the absence of profound baseline impairment, paralleling findings previously reported in healthy volunteer studies. It will be important for future studies to extend these findings to clinical populations characterized by more significant cognitive deficits, in order to further establish clinical relevance.

It is also important to note that this study involved a sub-acute dose of prucalopride (7–10 days). It remains possible that cognitive effects may differ with a longer dose regime. However, pre-clinical evidence indicates that 5-HT_4_ R agonism induces rapid increases in BDNF and promotes neuroplasticity, occurring substantially faster than the effects typically observed with SSRIs (Lucas et al., 2007; Mendez-David et al., 2014). This rapid action is thought to arise from selective receptor activation, which results in a near-immediate increase in firing of serotonergic neurons in the raphe nuclei (Faye et al., 2019). This pharmacological mechanism contrasts with that of SSRIs, as well as other pro-cognitive medications such as methylphenidate, which act by blocking presynaptic neurotransmitter reuptake and exert broader, less receptor-specific effects. Although direct head-to-head comparisons are lacking, prucalopride appears to share similarities with methylphenidate in healthy volunteers with respect to acute procognitive effects on processing speed, working memory, and verbal learning and memory (Aitken et al., 2025; Repantis, Schlattmann, Laisney, & Heuser, 2010). Acute procognitive effects have also been reported with modafinil, particularly in sleep-deprived individuals; however, these are less pronounced than its general effects on arousal, do not appear to persist over time, and are less consistently observed in healthy, well-rested individuals (Repantis et al., 2010). Finally, placebo participants guessed their allocation correctly more often than prucalopride participants, and thus, there may have been the potential for expectancy effects to be present to some extent.

In conclusion, this is the first study to assess the cognitive effects of 5-HT_4_R agonism in a remitted depressed population. We replicated and extended prior evidence from healthy participants, demonstrating a pro-cognitive effect of prucalopride across verbal memory, face processing, and working memory tasks. These effects appeared independent of emotional processing changes, highlighting a distinct neuropsychological profile compared with conventional antidepressants. People with remitted depression have a broad range of cognitive deficits, and it should be investigated whether targeted treatment of these with pro-cognitive medications may have beneficial effects on both prognosis and functioning. Future work should explore the therapeutic potential of 5-HT_4_R agonists in patients with depression and cognitive problems, investigate mechanisms of mood change outside of the emotional bias model, and further elucidate how 5-HT_4_ agonists may impact on the gut–brain axis. Given that cognitive deficits are an important feature of multiple serious mental illnesses, including schizophrenia and bipolar disorder, these findings may have broader transdiagnostic relevance.

## Supporting information

10.1017/S0033291726104450.sm001 De Cates et al. supplementary material De Cates et al. supplementary material

## Supplementary material

The supplementary material for this article can be found at http://doi.org/10.1017/S0033291726104450.

## Long descriptions

### Figure 1. Long description

Panel a is a flowchart titled Stimulus and Main response recorded. It shows three rows. The first row shows List A read aloud five times for trials 1 through 5, resulting in the number of List A words recalled correctly after each play. The second row shows List B read aloud once, resulting in the number of List B words recalled correctly. The third row shows delayed recall for List A at 2 minutes and 20 minutes with no new stimulus, represented by a large X, resulting in the number of List A words recalled correctly. A large downward arrow on the right indicates the chronological order.

Panel b is a line graph. The y-axis is Mean numbers of words recalled ranging from 8 to 14. The x-axis is Block with eight categories. List A T 1, List A T 2, List A T 3, List A T 4, List A T 5, List B, List A S D, and List A L D. A legend on the right identifies a grey line for Placebo and a blue line for Prucalopride. Both groups show a logarithmic-style increase from T 1 to T 5. The Prucalopride group consistently performs higher than the Placebo group across these five trials, marked by an asterisk indicating statistical significance. Both groups drop sharply at List B to approximately 8 words. For the delayed recall blocks S D and L D, both groups recover to between 11 and 12 words, with the Prucalopride group showing a slightly steeper decline between S D and L D compared to the Placebo group. Shaded areas and error bars represent the S E M.

Navigate back to Figure 1..

### Figure 2. Long description

Panel a shows a flowchart of the verbal N-back task. Four columns represent 0-back, 1-back, 2-back, and 3-back conditions. Each column shows a vertical sequence of letters in gray boxes connected by downward arrows, with a large white arrow on the right indicating the flow of 10 stimuli per block. Timing is noted as 1.5 s for stimulus and 1 to 2 s for intervals. Blue boxes at the bottom indicate target stimuli for a same response.

Panel b is a line graph showing Response time for correct hits in m s on the y-axis against Working memory load level n-back on the x-axis. Both the Placebo group in gray and the Prucalopride group in blue show a linear increase in response time from 0-back to 3-back. The Prucalopride group consistently maintains a lower mean response time than the Placebo group. An asterisk indicates statistical significance at the 3-back level.

Panel c is a line graph showing Accuracy for target hits in percentage on the y-axis against Working memory load level n-back on the x-axis. Accuracy for both groups remains high at 0-back and 1-back, then drops significantly at 2-back and 3-back. The Prucalopride group shows slightly higher accuracy than the Placebo group at the 1-back level.

Panel d is a scatter plot with regression lines showing Mean accuracy for target hits in percentage on the y-axis against Mean reaction time in m s on the x-axis. The Prucalopride group in blue shows a steep negative correlation, where accuracy decreases as reaction time increases. The Placebo group in gray shows a much flatter, nearly horizontal regression line, indicating a weaker relationship between speed and accuracy.

Navigate back to Figure 2..

### Figure 3. Long description

The figure consists of four panels labeled a through d.

Panel a is a flowchart of the F E R T task. It shows three vertical face images representing different emotions. An arrow labeled Identify the expression points to a central cross surrounded by eight gray boxes containing abbreviated emotion labels: D I S G, F E A R, H A P, S A D, S U R P, N E U T, A N G, and a central cross. Text indicates images are shown for 500 m s at 10 to 100 percent emotion or neutral.

Panel b is a bar graph of Mean accuracy in percent. The y-axis ranges from 0 to 100. The x-axis lists Anger, Disgust, Fear, Happy, Sad, and Surprise. Blue bars represent the Prucalopride group and gray bars represent the Placebo group. Accuracy is highest for Happy and Surprise and lowest for Fear. Individual data points are overlaid on the bars.

Panel c is a bar graph of Mean reaction time in m s. The y-axis ranges from 0 to 3000. The x-axis categories match panel b. Reaction times are generally higher for the Prucalopride group across all emotions, with the longest times recorded for Fear.

Panel d is a bar graph of the Number of misclassifications. The y-axis ranges from 0 to 80. The x-axis includes Neutral in addition to the six emotions. Misclassifications are significantly higher for the Neutral category compared to specific emotions, with the Placebo group showing a higher mean number of neutral misclassifications than the Prucalopride group, marked by an asterisk for statistical significance.

Navigate back to Figure 3..

### Table 1. Long description

The table is organized into five columns: Variable measure, Measurement type, a sub-category column, Placebo N equals 25, and Prucalopride N equals 24.

* Sex: Placebo is 36.0 percent Male and 64.0 percent Female. Prucalopride is 29.0 percent Male and 71.0 percent Female.

* Age: Mean in years S D is 30.2 9.95 for Placebo and 28.0 7.16 for Prucalopride.

* Years of education: Mean S D is 17.6 3.04 for Placebo and 17.8 2.63 for Prucalopride.

* First language: English is 72.0 percent for Placebo and 91.7 percent for Prucalopride.

* Ethnicity: Placebo is 56 percent White, 28 percent Asian, 4 percent Black, and 12 percent Other or mixed. Prucalopride is 50 percent White, 16.7 percent Asian, 8.3 percent Black, and 25 percent Other or mixed.

* Current smokers: 12.0 percent for Placebo and 16.7 percent for Prucalopride.

* Alcohol: Mean units per week S D is 2.53 3.43 for Placebo and 2.66 3.39 for Prucalopride.

* Caffeine: Mean cups per day S D is 1.53 1.35 for Placebo and 1.30 1.10 for Prucalopride.

* Body mass index: Mean S D is 24.7 3.63 for Placebo and 23.4 3.25 for Prucalopride.

* Systemic contraception: 28 percent for Placebo and 13 percent for Prucalopride.

* Depression P H Q 9: Mean S D is 3.08 2.71 for Placebo and 2.38 2.24 for Prucalopride.

* Subjective cognition P D Q 20: Mean S D is 25.3 11.8 for Placebo and 19.4 11.7 for Prucalopride.

* Anhedonia S H A P S: Mean S D is 0.60 1.63 for Placebo and 0.50 0.83 for Prucalopride.

* Anxiety S T A I T: Mean S D is 33.6 8.7 for Placebo and 38.0 10.1 for Prucalopride.

* Affect P A N A S: Positive mean S D is 31.3 6.57 for Placebo and 33.8 7.62 for Prucalopride. Negative mean S D is 12.7 3.12 for Placebo and 11.8 2.26 for Prucalopride.

Navigate back to Table 1..
